# An unusual presentation of prominent crista terminalis mimicking a right atrial mass: a case report

**DOI:** 10.1186/s12872-018-0925-y

**Published:** 2018-11-07

**Authors:** Jiang Wang, Gang Wang, Xiao Bi, Ran Zhang, Changfu Liu

**Affiliations:** 1 Department of Pulmonary & Critical Care Medicine, General Hospital of Chinese People’s Liberation Army, 28th Fuxing Road, Beijing, 100853 China; 2 Department of Cardiology, Anshan Central Hospital, 77th Zhonghua Road (South), Anshan, 114001 Liaoning Province China; 3 Department of Nuclear Medicine, General Hospital of Chinese People’s Liberation Army, 28th Fuxing Road, Beijing, 100853 China; 4 Department of Cardiology, General Hospital of Chinese People’s Liberation Army, 28th Fuxing Road, Beijing, 100853 China

**Keywords:** Crista terminalis, Right atrial mass, PET/MRI, Echocardiogram

## Abstract

**Background:**

The crista terminalis is a variation of normal anatomical structure within the right atrium which may be misdiagnosed with an abnormal atrial mass normally visualized in the standard views on the transthoracic echocardiogram.

**Case presentation:**

In this case presentation, we demonstrated a rare case report describing the accidental discovery of a right atrial mass-like structure in a 54-year old Asian man without physical discomfort during an echocardiographic examination. These findings naturally caused some concern as the differential diagnosis such as right atrial myxoma or thrombus and further examination were organized. The subsequent positron emission tomography/magnetic resonance imaging (PET/MRI) differentiated a true right atrial mass from a strip extending into the atrium in accordance with prominent crista terminalis.

**Conclusion:**

A preferable understanding of the complex anatomy and PET/MRI appearance of a prominent crista terminalis will minimize the misdiagnosis of this structure and avoiding unnecessary anxiety and more invasive examinations.

**Electronic supplementary material:**

The online version of this article (10.1186/s12872-018-0925-y) contains supplementary material, which is available to authorized users.

## Background

A prominent crista terminalis is a well-defined fibromuscular ridge formed by the junction of the sinus venosus and primitive right atrium (RA) extending along the posterolateral aspect of the right atrial wall, which is a normal anatomic variant and recognized by echocardiography occasionally [1, 2]. However, this variant structure can be misdiagnosed with true right atrial mass like tumor, thrombus or vegetation [3–11]. Compared with the method in echocardiography, the ability of cardiac positron emission tomography/magnetic resonance imaging (PET/MRI) to tissue characteristics enables differentiation between tumor, thrombus, structural abnormalities and normal variant anatomical structures, which is frequently recommended for further evaluation in presence of an unclear cardiac mass and minimizes the misdiagnosis of variant structure [12–14]. In this case report, we present a case of a cardial mass within right atrium on echocardiography, which is differentiated from a pathological cardiac mass by multiplanar imaging of cardiac MRI, enhancement and ^18^F-FDG uptake of tissue for understanding of the complex anatomy of the heart and its anatomical variations. An appreciation of the anatomy and MRI appearance of the crista terminalis will minimize the misdiagnosis of this structure and avoid additional unnecessary and more invasive tests.

## Case presentation

A 54-year-old man was admitted to our hospital for a mass in RA on an echocardiography examination occasionally. There was no symptom or sign of fever, chest pain, dizziness, palpitations and no history of a heart disease and tumor. He was a teacher, running and training on a weekly basis. He also was a non-smoker and drank 50 ml Chinese liquor each day for 30 years. There was no other medical history of note and no family history of sudden death. Clinically, the patient has stable vital signs with no fever, a heart rate of 68 beats per minute and blood pressure of 128/79 mmHg. The serum tumor markers and D-dimer were normal. An electrocardiogram (ECG) showed normal sinus rhythm. The chest X-ray showed normal cardiac size and clear lungs.

**Fig. 1:**
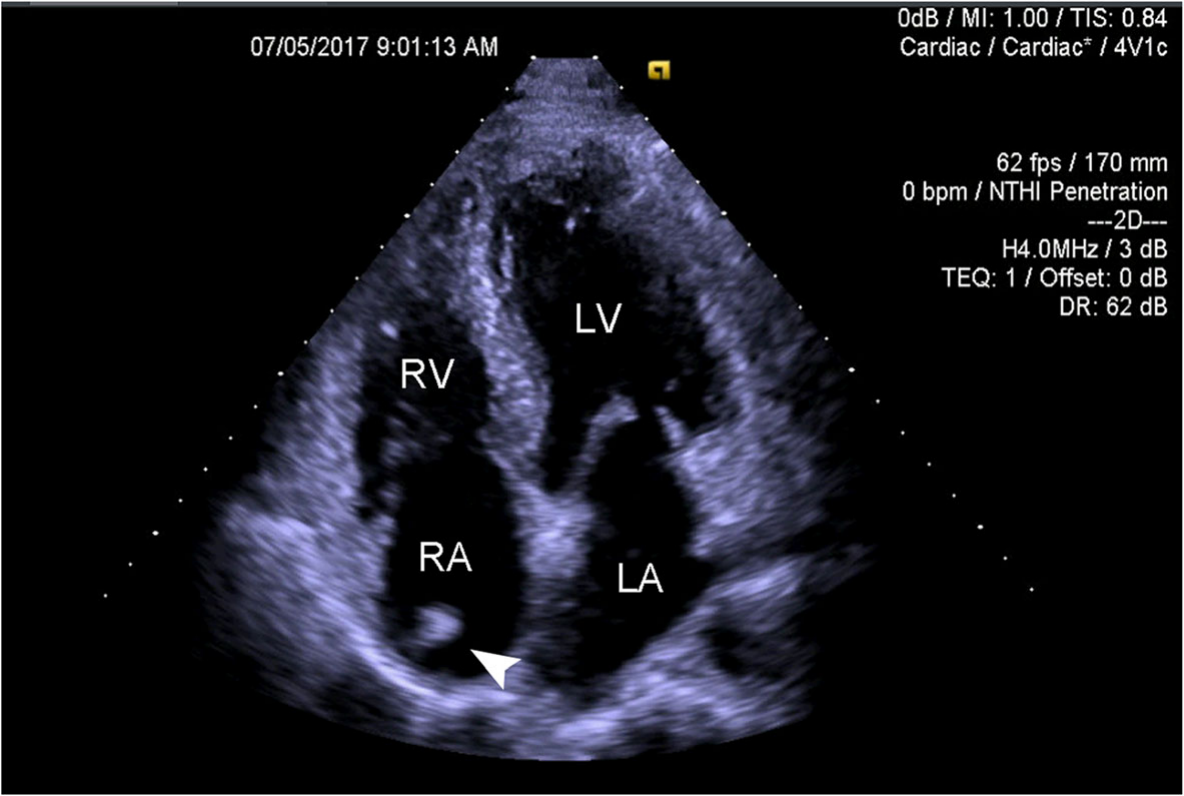
Transthoracic echocardiogram, in four-chamber apical view, showed an echogenic mass (arrowhead) in the right atrium. LA: left atrium, LV: left ventricle, RA: right atrium, RV: right ventricle

The echocardiography (Siemens, ACUSON SC2000) showed normal left ventricular systolic function (ejection fraction 66%), normal left and right ventricular cavities and normal cardiac valves. Only mild tricuspid and aortic valve regurgitate. Incidentally, an apparently smooth mass-like echogenic structure (9*11 mm) attached to tricuspid valve was noted in the right atrium and suggestive of a thrombus or a tumor in four-chamber apical view (Fig. 1).

**Fig. 2:**
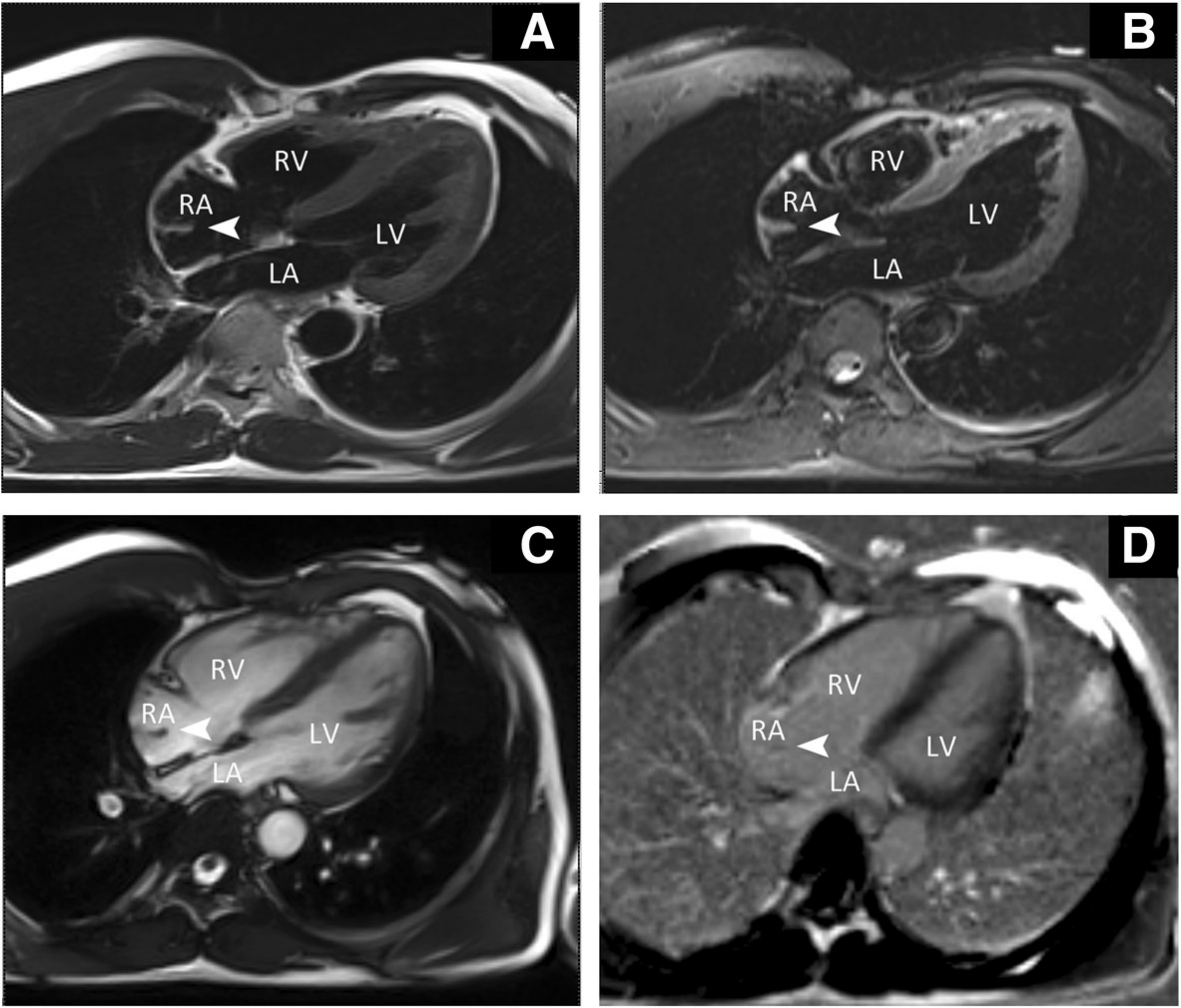
Four chamber view. **a**. On T2-STIR image, the mass (arrowhead) had similar signal intensity to myocardium revealing a myocardial structure and excluding lipoma. **b**. On T2-STIR with fat suppression image, the consequence is consistent with (**a**). **c**. The cine MRI confirmed a mass (arrow-head) attached to the posterior wall of the right atrium. D. LGE-sequences indicated no abnormal enhancement of the mass (arrow-head). T2-STIR, T2-weighted short tau inversion recovery; LA, left atrium; LV, left ventricle; RA, right atrium; RV, right ventricle; LGE, late gadolinium enhancement

**Fig. 3:**
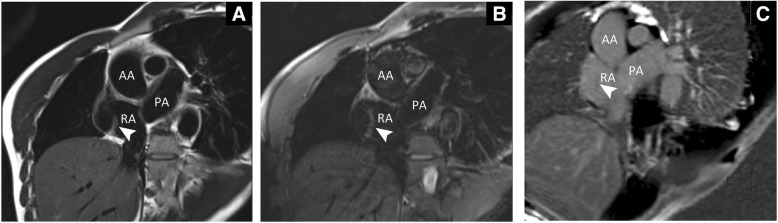
Short axis view. **a**. On T2-STIR image, the mass (arrowhead) had similar signal intensity to myocardium revealing a myocardial structure and excluding lipoma. **b**. On T2-STIR with fat suppression image, the consequence is consistent with (**a**). **c**. LGE-sequences indicated no abnormal enhancement of the mass (arrowhead). T2-STIR, T2-weighted short tau inversion recovery; RA, right atrium; PA, pulmonary artery; AA, ascending aorta; LGE, late gadolinium enhancement

**Fig. 4:**
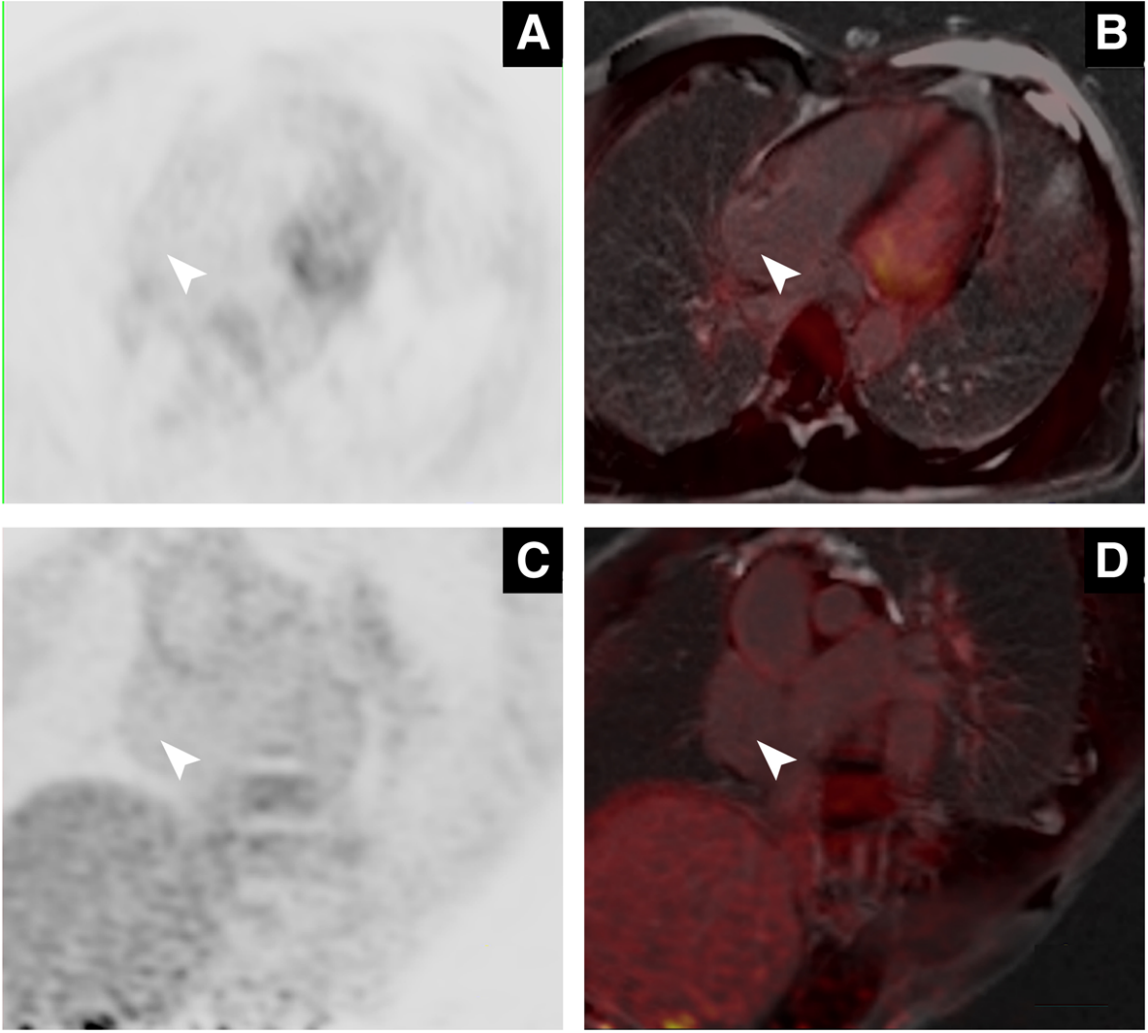
The four chamber view of the heart using PET (**a**) and fused PET/MRI (**b**) demonstrated no obvious radiotracer uptake on the mass (arrowheads). Short axis view of the heart using PET (**c**) and fused PET/MRI (**d**) also indicated nothing unusual on the mass (arrowheads)

Due to the limitation of acoustic window of echocardiography, the patient was arranged to our center of nuclear medicine for PET/MRI for diagnostic work up of the mass to be determined. Cardiac PET/MRI was performed with Siemens Biograph mMR (Software version B20P, Siemens Healthcare, Erlangen, Germany). The MRI showed the findings: Cine gradient-echo image in four-chamber view confirmed the presence of a banded structure (arrowhead) attached to the posterior wall of RA and the mass moved during systole and diastole period (Fig. 2c) (See Additional file 1: Video S1). On T2-weighted short tau inversion recovery (T2-STIR) of the four chamber and short axis view (Figs. 2a and 3a), the mass (arrowhead) had similar signal intensity to myocardium revealing a myocardial structure. On T2-STIR with fat suppression of the four chamber and short axis view (Figs. 2b and 3b), the results were consistent with T2-STIR images. There was no abnormal enhancement about the mass (arrowhead) on late gadolinium enhancement (LGE)-MRI (Figs. 2d and 3c). Furthermore, PET/MRI indicated no obvious ^18^F-fluorodeoxyglucose (^18^F-FDG) uptake on the mass (Fig. 4, arrowhead), which exclude malignant tumor. These typical findings detected by PET/MRI lead to the diagnosis of a prominent crista terminalis. The case follow-up hitherto had no special symptom.

## Discussion

The crista terminalis is a landmark of the RA, extending along the posterolateral aspect of the right atrial wall. It is the fibromuscular ridge begins at the upper portion of the septal surface and passes anteriorly to the opening of the superior vena cava and terminates at the lateral side of the entrance of the inferior vena cava. When the embryo developed to sixth weeks, the right angle of the venous sinus, the superior inferior vena cava and the original atrium were fused to form the crista terminalis. It divided the RA into two sections: atrium proper and sinus venarum cavarum and corresponded to the sulcus terminalis of the surface of right atrium [15, 16]. On microscopic view, the crista terminalis consists of three cells: working cells, pacemaker cells and transitional cell, of which the myofibers consist of two layers; the deep is thick and the superficial is only in the middle to lower part of crista terminalis. The pacemaker cells play no role in sinus pacemaking and conduction. While in the majority of patients, the crista terminalis is a normal and benign mass. The changes of velocity and direction of conduction result in cardiac arrhythmia on pathophysiological condition and the anatomic importance of crista terminalis related to arrhythmia is well documented, which has been studied most extensively by electrophysiologists. The crista terminalis can initiate ectopic atrial beats, especially right atrial tachyarrhythmia, which lead to paroxysmal atrial fibrillation or atrial flutter [16]. Sanchez-Quintana et al. [17] found that two-thirds of focal right atrial tachycardia, in the absence of structure heart disease, arise from the crista terminalis. In our case report, the patient has no clinical symptoms and the ECG is normal. We can recommend him review regularly and visit periodically.

The differential diagnosis of the crista terminalis is particularly important, especially the mass in the RA, which is mostly detected by echocardiography except for the clinical manifestations of primary diseases. The tumor, thrombus and vegetation are the common cardial mass. And the tumor according to source are divided into primary tumors and metastases [18, 19]. So the early detection and accurate identification of these mass are of great significance in the diagnosis and prognosis. Except these actual mass, there are many right atrial structures which can mimic an abnormal mass and may not be shown well on routine standard views by echocardiography. These structures included the Eustachian valve, thebesian valve, persistent sinus venosus, Chiari network and the crista terminalis [20]. In this case, we misdiagnose the mass as tumor or thrombus on echocardiography initially and finally diagnose it as crista terminalis by other subsequent measures. It is significant for us to take full advantage of multiple methods and make comprehensive analysis on cardial mass-like structure.

The detection of the cardial mass is mainly based on echocardiography, computed tomography (CT), and cardial MRI. Echocardiography is the first choice for the detection and evaluation of cardiac space occupying lesions [21, 22]. Among them, two-dimension can helps reveal mass location, size, mobility and differentiation from extracardiac disease. And doppler echocardiography can evaluate the hemodynamic changes caused by cardiac mass, which are generally not needed in the atrium. When the heart is examined by echocardiography, we should pay attention to direct and indirect signs. The direct signs are the abnormal echo lump in the cardiac cavity. We should know its morphology, the relationship with the heart wall, activity, hardness and echo intensity. The mass effect mainly depends on the location of the tumor. The intracavitary tumor of the right atrium mainly causes the enlargement of the right atrium, the decrease of the pulmonary blood and the dilatation of the superior vena cava, similar to the mitral valve damage or the constrictive pericarditis. The high pressure signs of pulmonary circulation, which are mainly pulmonary veins, are important indications of left atrial tumor. Evaluation with echocardiography may be limited in patients with a large body habitus. Transesophageal echocardiography, with the help of esophagus probe placed in the esophagus or stomach fundus, can scan the heart from back to front. It provide better spatial resolution, high imaging quality and can be used to differentiate non-pathologic structures from pathologic ones in right atrial structure, which contributing to diagnosis better [2, 23], but restricted by its invasive and not as widely available.

In recent years, multi-slice spiral-CT and MRI has been the next strategies of choice after echocardiography, which can provide definite diagnosis evidence for cardiac tumors depending on their dynamic images, high resolution, multiple plane reconstruction and the sensitivity of calcification and fat within a mass. It is of great help to the diagnosis of the lesions property, location, the scope of the invasion and the identification of the pericardial and mediastinal tumors [24]. CT scan described the crista terminalis as a band extending obliquely across the right ventricle [25]. Cardiac MRI scan described the crista terminalis as the line of union between the RA and the right auricle, which is in signal intensity to myocardium. Gadolinium contrast material is helpful in differentiating a thrombus or crista terminalis from tumor, as the former does not show enhancement [7]. However, we can’t ignore the negative impact and contraindication such as risks from contrast use, radiation and patients with claustrophobia, pacemaker or other metallic implants. The choice of technique to use after echocardiography still depends on the actual situation and suggestion of clinician.

PET is a positron emission tomography technology which using the radiolabeled glucose analogue ^18^F-FDG as a tracer is a functional image technique used to differentiate metabolic changes in normal cells and malignant cells with a carcinoma of unknown origin. Compared with normal tissues, the tumor cells can take up ^18^F-FDG more than several times and obtain this information through PET imaging [26, 27]. It has been a new imaging technique applied to the clinic after CT and MRI. With the development of science and technology, MRI has been combined with PET representing an exciting technological advance, which provides not only functional and metabolism images but also morphologic information especially soft tissues [28]. In this case report, there was no obvious 18F-FDG uptake about the mass on PET and PET/MRI images, by means of Biograph mMR PET-MR system, which help us exclude malignant tumor more accurately. PET/MRI as an integrated imaging technology would allow simultaneous data acquisition, resulting in combined functional and morphological images with an excellent soft tissue contrast, good spatial resolution of the anatomy and accurate temporal and spatial image fusion with a broader prospect of development. However, there are some limitations for PET scan. As for benign mass such as atrial myxoma, a usual benign tumor, PET/CT scan usually presents no obvious FDG uptake, which usually is not different to normal heart cells. PET/CT scan usually has application value in differentiating malignant and benign lesions. While echocardiography has characteristic sign in atrial myxoma. To any uncertain cardial mass, we should depend on multiple detection methods and comprehensive judgment rather than focusing on single methods.

Endomyocardial biopsy (EMB) usually plays a pivotal role in the diagnosis of some diseases, which is the “gold standard” by providing heart tissue in vivo [29]. The diagnostic level of this tool is cellular and it can provide specific etiologic information for therapy and management. In our case, by comprehensive analysis of the above results, the diagnosis is specific. The EMB can be crucial for special cases diagnosed difficultly.

In addition, there were a few case reports of the prominent crista terminalis in the literature, in retrospect, diagnosed with different tools and interestingly all of them are female (Table 1). However, the case report we presented was male and the clinical history and feature were non-specific. We should pay more attention to these case, try to summarize features and avoid misdiagnosis.

**Table 1:** Case reports on prominent crista terminalis

Author	Sex/Age	History	TTE	3D-TTE	TEE	CT	MRI	PET
D’Amato [4]	F/71	Hypertension AF	√		√			
Mckay [6]	F/49	Healthy	√	√				
Akcay [1]	F/51	Dyspnea Palpitations	√		√			
Gaudio [7]	F/68	Hypertension	√				√	
Pharr [9]	F/77	COPD Dyspnea	√		√			
Pharr [9]	F/74	Legs edema	√		√			
Pharr [8]	F/58	Dyspnea	√		√			
Alessandro [2]	F/26	ESRD	√		√		√	
Bannas [11]	F/57	Hypertension Dyspnea	√				√	
Jin [10]	F/73	TS Dyspnea PE	√		√	√	√	
Massimo [5]	F/64	Dyspnea Palpitations	√			√	√	
Salim [3]	F/32	Dyspnea Dizziness AF	√				√	
Present study	M/54	Healthy	√				√	√

Abbreviation: *AF* atrial fibrillation, *COPD* chronic obstructive pulmonary disease, *ESRD* end stage renal disease, *PE* pulmonary embolism, *TS* tuberculosis spondylitis

## Conclusion

In summary, our case report demonstrated that the crista terminalis is an unfrequent anatomy variation and example of pseudo-mass which can be easily misdiagnose for a mass. There is no doubt that the usefulness of TTE and TEE for the correct detection of prominent crista terminalis is certain. PET/MRI imaging remains a good alternative if a doubt remains after the TTE and TEE examination, which can make accurate diagnosis and avoid misdiagnosis and redundant invasive measures.

## Additional file

Additional file 1: Video 1. (Four chamber view of the heart using cine MRI): Cine MRI confirmed a mass (arrow-head) attached to the posterior wall of the right atrium. (AVI 833 kb)

## Abbreviations

AA: Ascending aorta
AF: Atrial fibrillation
COPD: Chronic obstructive pulmonary disease
CT: Computed tomography
ECG: Electrocardiogram
ESRD: End stage renal disease
LA: Left atrium
LGE: Late gadolinium enhancement
LV: Left ventricle
PA: Pulmonary artery
PE: Pulmonary embolism
PET/MRI: Positron emission tomography/magnetic resonance imaging
RA: Right atrium
RV: Right ventricle
T2-STIR: T2-weighted short tau inversion recovery
TS: Tuberculosis spondylitis
